# Analysis of alcohol policy in Nigeria: multi-sectoral action and the integration of the WHO “best-buy” interventions

**DOI:** 10.1186/s12889-019-7139-9

**Published:** 2019-06-24

**Authors:** Opeyemi Abiona, Mojisola Oluwasanu, Oladimeji Oladepo

**Affiliations:** Department of Health Promotion and Education, Faculty of Public Health, College of Medicine, University of Ibadan, Ibadan, Nigeria

**Keywords:** Alcohol, Health policy, Public health, Nigeria

## Abstract

**Background:**

Harmful alcohol use is a modifiable risk factor contributing to the increasing burden of non-communicable diseases and deaths and the implementation of policies focused on primary prevention is pivotal to address this challenge. Policies with actions targeting the harmful use of alcohol have been developed in Nigeria. This study is an in-depth analysis of alcohol-related policies in Nigeria and the utilization of WHO Best Buy interventions (BBIs) and multi-sectoral action (MSA) in the formulation of these policies.

**Methods:**

A descriptive case study design and the Walt and Gilson framework of policy analysis was utilized for the research. Components of the study included a scoping review consisting of electronic search of Google and three online databases (Google Scholar, Science Direct and PubMed) to identify articles and policy documents with no language and date restrictions. Government institution provided documents which were not online. Thirteen policy documents, reports or articles relevant to the policy formulation process were identified. Other components of the study included interviews with 44 key informants (*Bureaucrats and Policy Makers*) using a pretested guide. The qualitative data were coded and analyzed using thematic analysis.

**Results:**

Findings revealed that policy actions to address harmful alcohol use are proposed in the 2007 Federal Road Safety Act, the Non-communicable Diseases Prevention and Control Policy and the Strategic Plan of Action. Only one of the best buy interventions, (restricted access to alcohol) is proposed in these policies.

Multi-sectoral action for the formulation of alcohol-related policy was low and several relevant sectors with critical roles in policy implementation were not involved in the formulation process. Overall, alcohol currently has no holistic, health-sector led policy document to regulate the marketing, promotion of alcohol and accessibility. A major barrier is the low government budgetary allocation to support the process.

**Conclusions:**

Nigeria has few alcohol-related policies with weak multi-sectoral action. Funding constraint remains a major threat to the implementation and enforcement of proposed policy actions.

**Electronic supplementary material:**

The online version of this article (10.1186/s12889-019-7139-9) contains supplementary material, which is available to authorized users.

## Background

The health, social and economic costs of alcohol-related harm and diseases are well-documented [1–4] and over 3 million deaths have been attributed to alcohol intake every year [4]. In Nigeria, alcohol is the sixth leading risk factor contributing to most death and disability [5] and the alcohol-attributable deaths in both sexes for liver cirrhosis, road traffic accidents, and cancer in 2016 was 42,120; 15,365 and 4687 respectively [4]. Odueme et al., 2008 reported that drink driving accidents are the major causes of injuries and several deaths to Nigerians [6] and the country has one of the highest fatalities of road traffic accidents estimated at 21.4% per 100,000 populations every year [7] and other sequelae include the cost associated with damage of infrastructures and medical treatment [8].

Nigeria currently ranks 27th globally in respect of adults alcohol drinking (age 15+) in liters per year, making it one of the leading African countries in alcohol consumption [9]. These ranking fails to take into cognizance, the unrecorded production and consumption of illicit and locally made alcoholic beverages sold freely at various places within the society including institutions of higher learning [10]. It has been reported that alcohol is the most commonly used drug in Nigeria characterized by “heavy episodic drinking” [11]. Alcohol consumption by females in Nigeria has been on the increase and this has been associated with civilization, globalization and the expansion of women’s liberation in the country [12].

The World Health Organization urged countries to prioritize and accelerate the development of policies to tackle the risk factors for NCDs. Specifically, in 2010, the WHO proposed the Global Strategy to reduce the harmful use of alcohol as well as the 2008 and 2013 Global Action Plans for the prevention and control of NCDs [13–15]. These documents stipulate the adoption of multi-sectoral action (*explained as “actions undertaken by sectors outside the health sector, possibly, but not necessarily, in collaboration with the health sector, on health or health-related outcomes or the determinants of health”*) for the development of national policies and strategic plans to reduce the burden of NCDs. In addition, the WHO also recommended specific best buy interventions (*defined as “an intervention for which there is compelling evidence that is not only highly cost-effective but is also feasible, low-cost and appropriate to implement within the constraints of the local health system”*) which member states are expected to mainstream into their policies and implement [16]. Specific best buys for alcohol include tax increases, restricted access to retailed alcohol and ban on alcohol advertising. Member countries are expected to adopt these global strategies to develop national policies and guidelines for alcohol control using underlying principles such as multi-sectoral actions and integration of the WHO Best Buy interventions.

A number of Member States have developed national alcohol policies and implemented actions to decrease drink– driving prevalence, limit access to alcohol and implement restrictions on alcohol marketing [15]. This encouraging trend is expected to continue in light of the readiness to combat NCDs and their risk factors, including unsafe use of alcohol, as “a precondition for, an outcome of and an indicator of all three dimensions of sustainable development: economic development, environmental sustainability, and social inclusion” [15].

Nigeria has developed some alcohol-related policies however, the extent to which these addressed the recommended WHO “*Best Buy Interventions*” and utilized the multi-sectoral action (MSA) is unknown. This study is an in-depth analysis of alcohol-related policies in Nigeria, and the extent to which the WHO Best Buy interventions (BBIs) and multi-sectoral action (MSA) were used in the formulation of these policies.

## Methods

### Design

This manuscript presents the findings from Nigeria from a study titled “*Analysis for Non-Communicable Disease Prevention Policies in Africa (ANPPA)*”. The study design and data collection procedures have been published [17]. Descriptive case study design was adopted and guided by the Walt and Gilson framework of policy analysis. This framework focuses on four policy factors: (i) policy content which outlines how issues are formed and framed, and how it features on the agenda. It also details the policy objectives, actions, programs, targets and required resources; (ii) the policy actors and their influence on policy-making under various conditions; (iii) procedures used in building and implementing the policy; and (iv) the milieu which influences policy-making such as epidemiological and demographic transition, methods of economic and social and changes, financial and economic and policy, the political system and peripheral causes [18, 19].

#### Study site

The study was conducted largely in Abuja- Nigeria’s capital city.

#### Sample size

The aim of the qualitative study was to generate rich information on the phenomenon under study. Forty-four (44) policy actors identified from diverse sectors who should have participated in the development of the alcohol- inclusive NCD prevention policy were identified. This was done by weighing the importance of such bodies to NCD policy development. Additional ones identified during interviews with the index key informants constituted the sample size for the Key Informant Interviews.

### Sampling strategy/interview including inclusion/exclusion criteria and frequency of sampling strategy

The team identified 44 individuals from different sectors based on relative significance and potential roles in the formulation and implementation of the National NCD prevention policies. The study population for the Key informant interview comprised policy actors and bureaucrats who either contributed or should have participated in the NCD policy process. The range of sectors for policy formulation on NCDs as suggested by Meiro-Lorenzo et al., 2011 guided the identification and selection of potential interviewees [20]. We used a snowball sampling technique in which stakeholders identified after interviews would recommend additional individuals pertinent to the study. A total of 44 policy actors were interviewed (35 index key informants and an additional 9 identified through snowballing). There were 20 interviewees from diverse government sectors (*Information, Health, Sports and Youth, Education, Food regulatory agency, Women Affairs Trade and Investment, Labor, Finance, Legislature and Justice*); 9 from academic and research institutions; 2 from the food /hospitality industry, 6 from NCDs professional bodies, 1 each from a religious organization, civil society/ NGOs and international organization.

### Data collection method and processes

Data collection comprised a scoping review and key informant interviews among the relevant stakeholders.

#### Scoping review

A scoping review was conducted by two members of the team (OA and MO) and involved an electronic search of Google and 3 online databases (Google Scholar, Science Direct and PubMed) and Google using the following search terms: *multi-sectoral approach, policy, best buys,* alcohol, taxation, marketing or endorsement, sponsorship, information, warning, access, marketing and promotion *and Nigeria* to identify policy documents and articles written in English language with no date restriction. In addition, government ministries, departments and agencies were contacted to provide policy documents that were not online. There was a desk review by all members of the team (OA, MO, and OO) and analysis of extracted data from 13 documents (Three reports from an international organization, 6 published articles, 1 media publication and three national policy documents/acts) focusing on MSA and BBI. The three national policy documents/acts were the 2007 Federal Road Safety Commission Act [21], the 2013 National Policy and Strategic Plan of Action on Non-Communicable Diseases and 2015 National Strategic Plan of Action on Prevention and Control of NCDs [22, 23].

#### Key informant interviews

The key informant interview guide (see Additional file 1) was used to obtain perspectives of 44 policy actors and bureaucrats in public and private sectors who participated or should have contributed in the NCD policy process from the various sectors. Key themes explored the availability of alcohol policies, policy content, policy context, and actors in policy formulation, policy Implementation and financing mechanism. The guide was pre-tested by research assistants and finalized.

In respect to the data collection process, study participants from the different sectors were contacted in writing. This was followed by a telephone conversation that enables us to fix dates and times for each proposed interview. On the day of the interview, agreed upon venues were used and consent to conduct the interviews obtained from each participant at the commencement of the interview. The interview discussion was tape recorded based upon participant approval. Each interview lasted between 50 and 75 min. At the end of each interview, the names, addresses, and telephone numbers of potential stakeholders who are germane to the study were requested from those interviewed for inclusion in the list of interviewees. Those suggested were contacted and followed up for the interview. At the end of this process, a total of 44 interviewees of policymakers (*Bureaucrats and Policy Makers*) in the private and public sectors were conducted. The composition is shown in Table 1.

**Table 1:** Types of stakeholders interviewed

Respondent type	Total number
Government sectors	24
Health (9)	
Education (2)	
Trade and Investment (1)	
Labor (1)	
Justice (1)	
Information (2)	
Finance (2)	
Youth and Sports (2)	
Women Affairs (1)	
Food, Drug Administration and Control (2)	
Legislature (1)	
Research/Academic institutions (9)	9
International Organizations (1)	1
NCD Associations/Alliance (3)	3
Professional bodies/Associations (3)	3
Donors	
NGOs/Civil Society (1)	1
Hospitality Industry (1)	1
Food and Beverage Manufacturing Industry	1
Religious Body (1)	1
Total	44

#### Data extraction and analysis

Information from alcohol policy documents obtained was extracted and imputed into a matrix with the present data-showing name of the policy, Publication Year, Names of divisions/sector involved in policy formulation type of Best buy interventions present and extent of MSA.

Multi-sectoral action was determined by counting the sectors and their level of involvement in the policy formulation process. Multi-sectoral action was categorized as “low” if less than five relevant sectors were involved in the policy formulation process, “moderate” if five to nine and “high” if ten or more sectors participated in the process respectively.

Audio recordings of interviews were transcribed verbatim and analyzed using thematic analysis. The team coded the transcripts and entered it into NVIVO 10 using a coding structure based on the interview guide. Codes were afterward organized by theme.

After data analysis, the preliminary findings from the analyzed data were validated by presenting them to some interviewed stakeholders or their representatives and others who were not part of those interviewed including the press, NGO representatives on February 8, 2016, in Abuja, Nigeria. Participants of the preliminary dissemination forum were briefed and also requested to provide information on missing information and references that need to be included as well as seek necessary clarifications. At the end of the meeting, the findings were adjudged by participants to reflect the summary of the data in line with the objectives.

### Ethical considerations

Approval was given by the University of Ibadan /University College Hospital Ethical Committee, Nigeria in 2013 before the commencement of data collection. Written consent was obtained from all participants.

The information provided by research participants were kept confidential. To this end, each participant was assigned a unique identifier number and s/he was identified by it in all documents and the project database. Participants’ demographic data and ID number were kept in a locked cabinet and access to this file was restricted to authorized personnel.

## Results

### Nigeria status on the development of comprehensive alcohol control policies

According to 39 of the 44 interviewees, comprehensive policies to control the production, advertisement, marketing, and availability of alcohol in Nigeria in line with WHO recommendations are not available. Nigeria has participated in global meetings on alcohol control over the years but this has not translated into comprehensive policies on alcohol control. Rather, preliminary discussions are ongoing which might lead to policy formulation. This finding is substantiated by quotes from key informant interviewees as outlined below:

*“We have gone for international forum or fora on alcohol control…….. my boss had to go for that meeting, of course, we don’t have a policy, but he came back with some documents that we can adapt in Nigeria. As I speak to you now we have a proposal. We have done a proposal in the past couples of years but nothing was done about it, so of course, we’ve done the proposal again to call stakeholders together to develop a comprehensive policy on alcohol control and wide range of stakeholders will be involved. I don’t have the names of all the stakeholders but l knows that the alcohol companies will also be invited….” (Official from the Health sector*).

“*To be honest with you, do we really have policies on alcohol in this country? we don’ t. I can walk into any bar parlor any garden and if I want to take a carton of beer, nobody would prevent me from doing so. ... It’s not punishable, you are allowed to drink whatever quantity you want, ...., nobody restricts you, it’s a free thing. So, I don’t think the government has a policy on alcohol” (Official from the Finance sector*).

Though the country has not developed a comprehensive policy in line with the WHO global strategy to reduce the harmful use of alcohol, there are existing policy documents from health and non-health sector that proposes actions for alcohol control. These are the 2013 and 2015 National Policy and Strategic Plan of Action on Non-Communicable Diseases from the health sector [21, 22] and the Federal Road Safety Act (2007) from the safety and traffic management sector [24].

Beyond the Nigerian government, International Center for Alcohol Policies^1^ [ICAP] (a non- governmental organization recognized and sponsored by global alcohol producers) had worked with the Beer Sector Group of Manufacturers Association and the Advertising Practitioners Council of Nigeria to advocate for self-regulation [23].

### Policy content of existing policies and best buys addressed

Findings from the document review of the 2013 and 2015 National Policy and Strategic Plan of Action on Non-Communicable Diseases showed that six actions are proposed to limit the harmful use of alcohol. Specifically, they are–*“discourage alcohol use among women; prevent underage alcohol consumption; discourage binge drinking with consumption of toxic local brew; prevent consumption of illegally brewed and distributed alcoholic beverages; prevent driving or operating machinery under alcohol influence and identify and manage alcohol use disorders”* [21, 22]*.*

The only best buy interventions for Alcohol delineated in the documents is *restricted access to retailed alcohol to prevent underage alcohol consumption*. Other best buy interventions such as increased taxation and alcohol advertisement bans were not addressed.

The document review of the Federal Road Safety Act (2007) also revealed similar findings. This document was enacted by the National Assembly to guide and regulate the activities and functions of the Federal Road Safety Commission- a government paramilitary agency established in 1988 with the statutory functions of ensuring safety and traffic management in Nigeria. The Act only outlines actions aimed at promoting restricted access and availability of alcohol to counter drink-driving aside several other mandates for the agency. Only one of the WHO best buys- *restricted access to retailed alcohol*, was outlined in this document. Actions which relate to alcohol control in the Federal Road Safety Act (2007) are the prosecution or penalty for persons who “(*i) hawk or take alcoholic drink or hard drug within radius of 200 m to a motor park, motorcycle park or bus stop (ii) drive or attempt to drive on the road under the influence of drugs or alcohol*”. The proposed actions are in tandem with actions recommended in the World Health Organization global strategy to reduce the harmful use of alcohol [13].

### Multi-sectoral involvement

The extent of use of MSA for the formulation of the 2007 Federal Road Safety Act is low. Findings from the review of the 2009 FRSC Corporate Road map [25] that was developed to guide the implementation of the Federal Road Safety Act revealed that only listed seven organizations representing three sectors had input into the development of the road map. Three key sectors were the Finance and Administration (*Bureau of Public Enterprises, National Planning Commission, Budget Office of the Federal Government of Nigeria and Office of the Economic Adviser to the President*), regulatory bodies for emergency services and road safety (*National Emergency Management Agency and the Federal Road Safety Commission*), Legislature and Presidency (*Secretary to the Government of the Federation*).

This indicates the low level of participation of important sectors. A probable reason accounting for this is the paramilitary mode of operation of the institution. However, 2009 FRSC Corporate road map proposed outlined activities to involve other relevant sectors in the implementation of the Act [25]. Proposed stakeholders included Ministry of Health and Justice, the Chairman, Governors Forum, Secretary General of the Federation, representatives of the Nigerian Union of Road Transport Workers, Nigerian Union of Journalist, National Automotive Council, Ministry of Works, Ministry of Education, and Federal Republic of Nigeria Vice President. However there is no information on the constitution and functionality of the proposed committee of stakeholders.

The extent of use of MSA for the alcohol policy actions outlined in the 2013 National Policy and Strategic Plan of Action on Non-Communicable Diseases was originally very low. However, with the recommendation of the WHO, the committee that developed the policy document was expanded with a broader representation in 2015 and an increase in the number of sectors/organizations from 45 to 80. For the initial development in 2013, six sectors -*health, transport, research and academic, professional associations, civil society organization, and industry* were involved. This increased to 15 in 2015 with the formulation of the 2015 National Policy and Strategic Plan of Action on Non-Communicable Diseases. Specifically, these were *civil society organizations, industry, information, agriculture, finance, trade, women affairs, legislature, health, education, transport, academic and research, professional associations, regulatory agencies and the private sector*. Significant sectors which were not involved in the reconstituted committee but who had a role to play in the policy implementation include *Urban and Regional Planning, Sports; Environment and transportation unions.*

### Facilitators and barriers to use of MSA

The interviewees did not identify any major facilitating factors for MSA in the formulation of the 2013 and 2015 National Strategic Plan of Action on Prevention and Control of Non-Communicable Diseases policy, except for the participation of some sectors. Other factors which have potential to enhance MSA use were confirmed by some of the participants from the health and non-health sector such as empowerment through regulations and financial back-up, joint conference/workshops and training as reflected in the quotes below:

“*Government should empower them [Actors/government institution involved in implementing policy actions] with regulations and financial back-up*” (Representative of Professional Body, code 018)

…… *“should be having a joint conference or a summit whereby issues will be discussed to let them see the havoc or implications of the product or whatever they produce there are statistics to show the mortality rate coming out of the consumption of alcohol alright and the implication if they distribute or disseminate this information to people concerned and then they know when they sit themselves they will see the importance of what the government is saying or whatever the policy say about production”* (Academic and medical sector code 025)

However, the significant challenges associated with MSA use include poor understanding by stakeholders of the roles and contributions of different sectors to NCD prevention as well as the tussle between government ministries on which anchor sector is to assume leadership of the process.

### Implementation monitoring and evaluation status

Currently the status of alcohol policy implementation is extremely poor for both the FRSC Act and the 2015 National Policy on the Control and Prevention of Non-Communicable Diseases. According to 32 out of the 44 respondents, one of the major barriers facing the implementation of strong legislations and regulations was the relevance of alcohol as a social substance. The quote below highlights these points.

“*Well, alcohol is a social substance and many cultures permit the usage……….. so, it becomes a little bit difficult if there is no awareness about what to do and I think it had been the major factor limiting the implementation [of the FRSC act] which has been near zero*” [Representative of the Academic and Medical Sector code 027)

Aside from the failure of the government to strengthen systems and structures for alcohol control, other challenges identified by 23 of the interviewees include funding limitations and poor literacy, poor deployment of the law enforcement and regulatory agencies and the lack of a legislation to regulate the alcohol industry. The quote below succinctly illustrates this:

“... *of course, [we have] a few acts of parliament prohibit for example drunken driving. Outside that, I don’t know whether that act also prevents excessive alcohol use in public places … but I know that of course, we have not even implemented that one in the sense that we don’t have kits where we can check the blood levels of alcohol …, we don’t have them, Then of course for the alcoholic breweries in Nigeria that produce alcohol, I don’t see a proactive way to try and enforce\government policies [among them]*. *Some of them [the labeling of alcoholic drinks content] are not laws such as having extra information on their label- For example, alcohol is dangerous to your health and all those things, .... I have really not seen that like the tobacco ones, you know. I am not satisfied really with policies regarding alcohol in Nigeria. It is an area we need to push forward….” [Member of the House of Representative code 011]*

According to the views expressed by the respondents, many Nigerian cultures permit the use of alcohol thus constituting a limitation for the enforcement of the implementation of the FRSC act. Studies have also documented that the Alcohol Industry has leveraged on the influence of this permissive culture for alcohol consumption coupled with custom and traditions which permit its use as a promotion strategy in Nigeria [12, 26]. An example is the advertisement of Seamans Schnapps as a libation drink. Also on a similar note, Orijin, an herbal alcoholic drink was introduced and launched across Nigeria in the palaces of traditional rulers [26]. These findings reveal the significant challenge linked to the permissive cultural inclination for use of alcohol which has been identified as a challenge for the implementation of the 2007 FRSC act and a bane for the formulation of stronger legislation to curtail the unsafe use of alcohol in Nigeria.

As detailed in the 2007 Federal Road Safety Act, subsidy for the implementation of the 2007 Act proceedings is expected to be through the Federal Road Safety Commission and consists of any subsidy or fiscal allocation, Loans from the federal government and monies realized by way of gifts, fines, testamentary disposition or grants-in-aid. However, in reality, funding for these actions has been minimal.

According to interviewees from the health sector, the implementation of the alcohol component of the 2015 National Strategic Plan of Action on Prevention and Control of NCDs is yet to commence.

## Discussion

### Status of alcohol policy formulation in Nigeria

The World Health Organization has delineated urgent actions to guide countries’ efforts to reduce harmful alcohol ue, its associated health, and social consequences [13]. Unfortunately, Nigeria has not made much progress in articulating and implementing policies to decrease harmful alcohol use. According to the WHO Global status reports on health and alcohol, Nigeria has no comprehensive, stand-alone policy document to regulate the production, advertisement, availability and promotion of alcohol in line with WHO recommendations. Furthermore, there are no national and state level monitoring systems to track alcohol consumption and monitor its health and social consequences [9] despite the country’s contribution and approval of the declarations at the 2008 World Health Assembly [12].

### WHO inclusion “best buy” interventions in existing health and non-health sector policies with components for alcohol control

The alcohol component of the Strategic Plan and National Policy of Action on NCDs has been formulated and the only WHO “best buy” interventions addressed is “*restricted access to retailed alcohol specifically the prevention of underage alcohol consumption*”. Other best buy interventions such as prohibitions on alcohol marketing and tax increase were not addressed. Furthermore, the policy remains in limbo as the document, has not been published and disseminated. The actions proposed in the 2007 Federal Road Safety Act (*a non-health – sector legal act developed to reduce the occurrence of highway accident*) only address one of the best buy interventions on *limited access to alcohol* but there are no legal actions to control the activities of the Alcohol industry. This is a major gap and underscores the low political priority for the control of harmful alcohol use. There remains an urgent need for Nigeria to develop a comprehensive alcohol policy in line with the 10 proposed targets of WHO 2010 global strategy to reduce the harmful use of alcohol which also includes the WHO best buy intervention [13]. This has to be addressed through intense advocacy by significant stakeholders including the civil society and academia organizations if the country is to achieve a significant reduction in the social and health burden linked with the harmful use of alcohol.

### Multi-sectoral action to prevent harmful alcohol use

Th*e Global action plan for the prevention and control of non-communicable diseases 2013–2020* underscored the importance of a harmonized multi-stakeholder engagement for health [15], as a core principle pertinent to the achievement of national and global targets to decrease the burden of non-communicable diseases. Despite of its significance, the level of MSA for the alcohol policy formulation process in the reviewed overarching NCD prevention and control policy document is low as several relevant sectors relevant to the achievement of the health and non-health sector policies were not involved in the formulation procedure. Besides, best buy interventions such as increasing taxes and controlling alcohol marketing are not well reflected in the policies probably as a result of its potential for accelerating implementation results.

### Barriers to the formulation of alcohol control policy

Of note are the major barriers to the NCD policy development process especially the cultural milieu which permits alcohol use, low political priority for NCDs and poor understanding of MSA. The Nigerian cultural and traditional milieu is favorably inclined and perpetuates alcohol use and within this context, it is difficult for government agencies to formulate, implement and enforce strong alcohol control legislation. The alcohol industry leverages this favorable milieu by advertising alcohol in a cultural context; promoting it as a beverage associated with culture and celebration thus exacerbating the situation and unwittingly undermining the policy formulation and weakening the implementation processes. To address this, there is a need for a cultural re-orientation and strong policies that note the traditional and social relevance of alcohol but deformalize its harmful use.

Furthermore, Nigeria is beleaguered with myriads of health and development challenges and alcohol control does not top the priority of policy imperatives. Unfortunately, the neglect of the alcohol policy has an extensive history; successive Nigerian governments continue to avoid the regulation of harmful alcohol use [27]. To surmount this challenge, there is need to learn lessons from the relatively successful tobacco control effort in Nigeria which was characterized by the high level of multi-sectoral action and activism by civil society organizations, government and professional organizations [28].

Other barriers documented were overdependence on financial assistance and grants from donor organizations to fund alcohol policy formulation and implementation activities coupled with the minimal or absent of government budgetary allocation to support the process. A typical initiative which reflects Nigeria is overdependence on donor organizations for funding its alcohol control initiatives is the “drink responsibly” campaign by brewers. This initiative is championed and funded by brewers, multi-national alcohol industries and the International Alliance for Responsible Drinking (formerly the International Centre for Alcohol Policy [ICAP]). The initiative promotes moderate alcohol consumption rather than abstinence even among drivers. According to Dumbili et al, 2013, the intervention is a charade that distracts and undermines efforts at proposing stronger legislation for alcohol control in Nigeria [12].

This is a fall-out of the low priority for alcohol control in Nigeria, unless the issue of poor government funding is addressed strongly and consistently, the possibility of the alcohol policy gaining the required thrust is doubtful. This should be an issue on the agenda at global and national meetings on alcohol control. Innovative funding system should be introduced where donor organizations, financial and government institutions jointly donate to a central fund for alcohol control. This will ensure the accessibility and availability of funds for alcohol control implementation and policy development.

The team encountered some limitations during the conduct of this study. Firstly, all the policymakers especially those from donor organizations could not be reached due to the Ebola outbreak which occurred during the collection of the data. However, we were able to find stakeholders from the public and private sector as well as the health and non-health sector which presents an opportunity for a good mix of information collected. Secondly, the minutes of meetings could not be accessed which possibly will have supplied more helpful information on the scope of multi-sectoral action as well as the participation and involvement of actors in the policy processes. In addition, the interviewees had some challenges recalling all the issues which transpired during the policy formulation process. Thirdly, there is a limit to the use of the case-study method as the results obtained is not generalizable to other countries due to Nigeria’s distinctive ethnicity and political situation which outlines the framework (Context and content) of the policies. More studies should investigate the use of explanatory policy process theoretical frameworks such as the Multiple Streams or Advocacy Coalition Framework [29] which will ensure an in-depth analysis, evaluation and critique of the policy dynamics.

## Conclusions

There is presently no stand-alone, comprehensive policy to regulate the harmful use of alcohol. Low multi-sectoral action characterizes the alcohol-related policies formulated by the health and non-health sectors. Overdependence on donor funding and poor government budgetary allocation are major barriers to alcohol control in Nigeria.

Recommendations: The policy actions on alcohol prevention and control in the existing NCD policies should be reviewed and updated by the NCD committee in line with global recommendations proposed by World Health Organization (2010) global strategy to reduce the harmful use of alcohol [13] with emphasis on best buy interventions using MSA. Besides, legal acts should be formulated and ratified to regulate the activities of the alcohol industries.

## Additional file

Additional file 1: Key informants Interview Guide. (DOCX 14 kb)

## Data Availability

Transcripts and datasets can be provided to interested persons upon request to the corresponding author. This can only be used for non-commercial purposes which ensures that participants’ confidentiality is protected.

## Abbreviations

ANPPA: Analysis for Non-Communicable Disease Prevention Policies in Africa
APHRC: African Population Health Research Center
BBI: Best buy intervention
FMoH: Federal Ministry of Health
FRSC: Federal Road Safety Corps
IARC: International Agency for Research on Cancer
IDRC: International Development Research Center
MSA: Multi-sectoral action
NCD: Non-communicable Diseases
NGOs: Non Governmental Organizations
RTA: Road traffic accident
WHO: World Health Organization

## Footnotes

1 International Center for Alcohol Policies (ICAP) no longer exists and is now part of the International Alliance for Responsible Drinking (IARD).

## References

[1] Anderson P, Baumberg B (2006). Alcohol in Europe– public health perspective: report summary. Drug Educ Prev Policy.

[2] International Agency for Research on Cancer. IARC monographs on the evaluation of carcinogenic risks to humans, volume 96. Alcohol consumption and ethyl carbamate. Lyon: IARC; 2010. Available from: http://monographs.iarc.fr/ENG/Monographs/vol96/mono96.pdf. PMC4781168 21735939

[3] Shield KD, Parry C, Rehm J (2014). Chronic diseases and conditions related to alcohol use. Alcohol Res.

[4] World Health Organization. Global status report on alcohol and health, 2018. Geneva: World Health Organization WHO Press; 2018. https://apps.who.int/iris/bitstream/handle/10665/112736/9789240692763_eng.pdf;sequence=1.

[5] Institute of Health Metrics and Evaluation (2017). What causes the most death and disability combined?.

[6] Odueme S (2008). Nigeria: concerns over menace of heavy-duty trucks in Lagos.

[7] World Health Organization. Global status report on road safety 2018. World Health Organization; 2018. https://www.who.int/violence_injury_prevention/road_safety_status/2018/en/.

[8] Ogeleyinbo C (2015). Study of drink driving in Lagos from the perspective of law enforcement officers.

[9] World Health Organization. Global status report on alcohol and health, 2014. Geneva: World Health Organization WHO Press; 2014.

[10] Obikeze N, Obi I (2013). Alcohol and violence among undergraduate students of Anambra State University. Res J Organ Psychol Educ Stud.

[11] Gureje O, Degenhardt L, Olley B, Uwakwe R, Udofia O, Wakil A, Adeyemi O, Bohnert KM, Anthony JC (2007). A descriptive epidemiology of substance use and substance use disorders in Nigeria during the early 21st century. Drug Alcohol Depend.

[12] Dumbili. E. Changing patterns of alcohol consumption in Nigeria: an exploration of responsible factors and consequences. Med Sociol 2013;7(Issue 1). Available from http://www.medicalsociologyonline.org/resources/Vol7Iss1/MSo-Volume-7-Issue-1.pdf. Accessed 22 Feb 2017.

[13] World Health Organization. Global strategy to reduce the harmful use of alcohol, 2010. Geneva: World Health Organization WHO Press; 2010. 10.2471/BLT.19.241737 PMC7047030 32132758

[14] World Health Organization . 2008-2013 action plan for the global strategy for the prevention and control of noncommunicable diseases: prevent and control cardiovascular diseases, cancers, chronic respiratory diseases and diabetes. World Health Organization; 2009. http://www.who.int/iris/handle/10665/44009.

[15] World Health Organization. Global Action Plan for the Prevention and Control of Noncommunicable Diseases 2013–2020. Geneva: World Health Organization; 2013. [Google Scholar].

[16] World Health Organization (2011). From burden to “best buys”: reducing the economic impact of non-communicable diseases in low- and middle-income countries.

[17] Juma PA, Mohamed SF, Wisdom J, Kyobutungi C, Oti S (2016). Analysis of non-communicable disease prevention policies in five sub-Saharan African countries: study protocol. Arch Public Health.

[18] Walt G, Gilson L (1994). Reforming the health sector in developing countries: the central role of policy analysis. Health Policy Plan.

[19] Overseas Development Institute (2007). How can the analysis of power and process in policy-making improve health outcomes?.

[20] Meiro- Lorenzo M, Villafana TL, Harrit MN. Effective responses to NCDs: embracing action beyond the health sector: World Bank; 2011. Available from http://siteresources.worldbank.org/HEALTHNUTRITIONANDPOPULATION/Resources/281627-1095698140167/EffectiveResponsestoNCDs.pdf. Accessed 15 May 2015

[21] Federal Road Safety Commission (2007). Federal Road Safety Act.

[22] Federal Ministry of Health (2013). National policy and strategic plan of action on non-communicable diseases ( NCDs).

[23] Federal Ministry of Health (2015). National strategic plan of action on prevention and control of non-communicable diseases.

[24] International Center for Alcohol Policies (2011). ICAP, APCON host 2011 advertising stakeholders forum on responsible advertising and marketing.

[25] Federal Road Safety Commission (2009). Federal Road Safety Commission, Corporate Roadmap.

[26] Obot IS (2015). Africa faces a growing threat from neo-colonial alcohol marketing. Addiction.

[27] Oluwaniyi OO (2010). Oil and youth militancy in Nigeria’s Niger Delta region. J Asian Afr Stud.

[28] Oladepo O, Oluwasanu M, Abiona O (2018). Analysis of tobacco control policies in Nigeria: historical development and application of multi-sectoral action. BMC Public Health.

[29] Moloughney B (2012). The use of policy frameworks to understand public health-related public policy processes: a literature review.

