# Supplementary material for: Analysis of alcohol policy in Nigeria: multi-sectoral action and the integration of the WHO “best-buy” interventions
Source: BMC Public Health. 2019 Jun 24;19:810. doi: 10.1186/s12889-019-7139-9 (PMC6591910; doi:10.1186/s12889-019-7139-9)
Supplement: Supplementary file 1 — Key informants Interview Guide. (DOCX 14 kb) [file 12889_2019_7139_MOESM1_ESM.docx]

### Key informants Interview Guide

**Introduction**

This study aims to understand how policies have been formulated and implemented to prevent non-communicable diseases in the country. The major non-communicable diseases we are focusing on include cardiovascular disease, cancers, chronic respiratory diseases and diabetes.

The preventive activities include tackling major risk factors and individual-based interventions for managing people at high risk of cardiovascular events. In particular, we would like to understand who is involved with non-communicable disease policy prevention policy development and implementation. In terms of policy, we are interested in higher level policies such as national plans, as well as lower level policies or guidelines related to NCD prevention and program strategies.

**Share the study information sheet and request for signed consent**

**Demographics, TAKE NOTES** (identifying information to be kept separate from interview transcripts)

Just to confirm that I have your details right…..

1. Participant’s name & organization and email/ contact details (fill in beforehand if possible):
2. Participant’s title/designation and primary responsibilities:
3. What year did you start working in this organization? What year did you start in this particular position?

**REQUEST TO TURN ON RECORDERS AT THIS POINT IN THE INTERVIEW**

1. **My first set of questions is about policies related to alcohol use.**

*[Alcohol policy]*

- 1. **[Provide interviewee in advance with summary of policies/implementation activities found through desk review; show the list here].** When you think about these policies [laws, regulations, documents, guidelines] that apply to the use, trade and advertisement of alcohol in (mention country), are there any that we are missing or in progress? [Note to interviewee; Prepare in advance so you know the policies]

*[Probe for: Guidelines, protocols, action plans, strategic frameworks, programmes and other documents]*

- 1. Please explain to me who was involved in formulating and implementing these alcohol policies?

*Probe for: Which ministries, heads, NCD heads, Civil societies, parliamentarians, manufactures, industries, farmers, etc.? How/when were they involved? Who else should have been involved?*

- 1. What factors helped people in formulating and implementing alcohol policies?

*Probe: Different for formulating policies vs. implementing programs? What were facilitators in past if there are policies in progress, what do you see now/in future?*

- 1. What do you think were the challenges [barriers] in involving different people/sectors in formulating and implementing alcohol policies?

*Probe: Different for formulating policies vs. implementing programs? What were challenges in past if there are policies in progress, what do you see now/in future?*

- Ask how has involving other actors facilitated things?
- How has it slowed down things?
- Mention the biggest achievement from having many actors involved
- Mention the biggest loss from having many actors involved
- What is the greatest learning from having many actors involved?
  1. What would you recommend to facilitate the working of different sectors in formulating and implementing alcohol policies for the future?

*Probe: Different for formulating policies vs. implementing programs? Strategies and suggestions.*

**Thank you for your time. This is the end of our discussion on today.**
